# Integrated Analysis of MicroRNA and mRNA Expression Profiles in the Fat Bodies of MbMNPV-Infected *Helicoverpa armigera*

**DOI:** 10.3390/v15010019

**Published:** 2022-12-21

**Authors:** Zhenpu Liang, Yanqing Yang, Xiaoyan Sun, Junyang Du, Qiuyun Wang, Guozhi Zhang, Jiran Zhang, Xinming Yin, Deepali Singh, Ping Su, Xiaoxia Zhang

**Affiliations:** 1 College of Life Sciences, Henan Agricultural University, Zhengzhou 450002, China; 2 College of Plant Protection, Henan Agricultural University, Zhengzhou 450002, China; 3 School of Biotechnology, Gautam, Buddha University, Greater Noida 201312, India; 4 Institute of Agricultural Sciences of the 14th Division of Xinjiang Production and Construction Corps, Kunyu 848116, China

**Keywords:** microRNA, MbMNPV, *Helicoverpa armigera*, RNA-seq, sfr-miR-6094-5p, eIF3-S7, host–virus interaction

## Abstract

MicroRNAs (miRNAs), are a novel class of gene expression regulators, that have been found to participate in regulating host–virus interactions. However, the function of insect-derived miRNAs in response to virus infection is poorly understood. We analyzed miRNA expression profiles in the fat bodies of *Helicoverpa armigera* (*H. armigera*) infected with *Mamestra brassicae* multiple nucleopolyhedroviruses (MbMNPV). A total of 52 differentially expressed miRNAs (DEmiRNAs) were filtered out through RNA-seq analysis. The targets of 52 DEmiRNAs were predicted and 100 miRNA–mRNA interaction pairs were obtained. The predicted targets of DEmiRNAs were mainly enriched in the Wnt signaling pathway, phagosome, and mTOR signaling pathway, which are related to the virus infection. Real-time PCR was used to verify the RNA sequencing results. ame-miR-317-3p, mse-miR-34, novel1-star, and sfr-miR-6094-5p were shown to be involved in the host response to MbMNPV infection. Results suggest that sfr-miR-6094-5p can negatively regulate the expression of four host genes eIF3-S7, CG7583, CG16901, and btf314, and inhibited MbMNPV infection significantly. Further studies showed that RNAi-mediated knockdown of eIF3-S7 inhibited the MbMNPV infection. These findings suggest that sfr-miR-6094-5p inhibits MbMNPV infection by negatively regulating the expression of eIF3-S7. This study provides new insights into MbMNPV and *H. armigera* interaction mechanisms.

## 1. Introduction

*Helicoverpa armigera* (*H. armigera*) is a worldwide pest [1] that damages several crops, such as maize, wheat, cotton, pepper, and tomato, and causes heavy losses [2]. *Baculoviridae* family comprises arthropod-specific viruses with double-stranded DNA genomes of 80–180 kb [3]. *Mamestra brassicae* multiple nucleopolyhedrovirus (MbMNPV) is a member of *baculoviridae* family, which is characterized by a wide host spectrum and known to be effective in the field control of *H. armigera* infection. Bioassay shows that MbMNPV has great application and has been developed as a commercial bio-pesticide in China [4]. However, not much is known about the molecular mechanism underlying the MbMNPV–host interactions.

RNA interference (RNAi) is the process by which double-stranded RNA specifically silences the expression of its homologous genes through the degradation of their cognate mRNA [5]. RNAi has shown enormous potential to be developed as a novel pest control strategy [6]. MicroRNAs (miRNAs) are a class of small non-coding RNAs that have proven roles in the regulation of gene expression by binding to the transcripts of targets, which can be pivotal in disease and pest control as well as virus–insect interactions [7]. Studies have found that the overexpression of Dicer-2 and Ago-2 genes can improve the cell defense ability against the acute infection of the cricket paralysis virus (CrPV) [8]. Another study reports Ecdysis of *Nilaparvata lugens* fed with nlu-miR-173 agomirs was compromised, which affected their normal growth [9]. Another report shows miR-14 positively regulates the *H. armigera* ecdysone receptor (Ha-EcR) during single nucleopolyhedrovirus (HaSNPV) infection. Consistent with decreased levels of miR-14 following HaSNPV infection, the transcript levels of its target gene Ha-EcR declined, which indicates that miR-14 is involved in the regulation of *H. armigera* growth and is related to virus–host interactions [10]. In conclusion, miRNAs play key roles in the growth of insects and offer an approach for controlling insect pests by inhibiting the growth and development of the pests [11].

The fat body of insects is a vital organ for biosynthesis, metabolism, detoxification, and control of insect ecdysis. It has become a significant research model for the study of metabolic disorders [12]. In this study, the changes in miRNA levels in the fat body of *H. armigera* in response to MbMNPV infection were investigated by the use of high-throughput sequencing. Our results proved insights into the role of miRNAs in *H. armigera*–MbMNPV interactions, which help us to further understand host–baculovirus interactions and provide new strategies for insect pest control by miRNA.

## 2. Materials and Methods

### 2.1. Insects and Viruses

The eggs of *H. armigera* were purchased from Jiyuan Baiyun Industry Co., Ltd. (Jiyuan, China). Insects were fed with an artificial diet and reared in the laboratory at 27 ± 2 °C, 60 ± 2% relative humidity with a photoperiod of 16:8 (light:dark) [13].

MbMNPV was provided by our laboratory and later propagated by feeding to third instar *H. armigera*. Occlusion bodies (OBs) of MbMNPV were purified as described by Kyei-Poku and Kunimi [14]. The OBs were purified from MbMNPV-infected cadavers by differential centrifugation followed by sucrose density gradient ultracentrifugation.

### 2.2. Bioassay and Sample Collection

The newly molted fourth-instar larvae with weights ranging from 0.32 g to 0.37 g, were fed with an artificial diet contaminated with MbMNPV occlusion bodies (OBs; 1 × 10^8^ OBs/larva) after starving for 20 h. Upon consumption of the diets, the larvae were fed with fresh diets without exposure to any pathogens. Mock-infected larvae were fed with the same quantity of diets, but coated with distilled water. Twenty-five larvae per group were evaluated for larval weight at 24, 48, and 72 h after infection with MbMNPV. The fat body was collected at 72 h after infection with MbMNPV. Larvae were anesthetized on ice for 30 min, the upper epidermis of the larvae was cut off, and the fat body was collected into a 1.5 mL Eppendorf tube on ice.

### 2.3. Identification of H. armigera Infected with MbMNPV

The copy number of MbMNPV was calculated by absolute quantification. In short, MbMNPV genomic DNA was extracted from the liquefied larvae infected with MbMNPV and used as the template for the amplification of the conserved polyhedrin gene by PCR. The PCR products were sub-cloned into the pMD™19-T Vector Cloning Kit (TaKaRa). The recombinant plasmid polyhedrin-pMD-19T was used to calculate the plasmid copy number and generate a standard curve for RT-qPCR. CT value was obtained by RT-qPCR using the specific primer of polyhedrin for the fat body of mock- and MbMNPV-infected larvae for 72 h, and then the copy number of MbMNPV was determined by standard curve. The *β-actin* gene was used as an endogenous reference. The primers were synthesized by Sangon Biotech Co., Ltd. (Shanghai, China) (Table S1).

### 2.4. RNA Extraction and Quality Control

Mock-infected and MbMNPV-infected larvae consisted of three repeats labeled as CK-1, CK-2, and CK-3 and MbMNPV-1, MbMNPV-2, and MbMNPV-3, respectively. At 72 h post-infection (hpi), total RNA was extracted using the mirVana miRNA Isolation Kit (Ambion) according to the manufacturer’s protocol. The RNA quality and purity were evaluated using agarose gel electrophoresis, Nanodrop microvolume spectrophotometer, and Agilent 2100 Bioanalyzer.

### 2.5. Library Preparation and Sequencing

Small RNA sequencing libraries were generated using the NEBNext Small RNA Library Prep Set for Illumina Kit (Cat. No. NEB#E7330S, NEB, USA) following the manufacturer’s recommendations. Briefly, 1 μg of total RNA was ligated with the 3′ adaptor and 5′ adaptor at each end. After reverse transcription, PCR amplification was performed and the products with fragments ranging from 140 to 160 bp in length were gel purified. Library quality was assessed on the Agilent Bioanalyzer 2100 system using DNA high-sensitivity chips. The library preparations were finally sequenced using the Illumina Novaseq 6000 platform.

### 2.6. MiRNAs Sequencing and Analysis

Sequencing data were analyzed by removing adaptor dimers, common RNA families (rRNA, scRNA, Cis-reg, snRNA, tRNA), and the sequences with repeats and low complexity by using Bowtie [15]. Subsequently, the sequences (15–41 nt) were mapped to the *H. armigera* genomic database against the miRBase v22 database [16] (http://www.mirbase.org/, August 2021) to identify known miRNAs and novel 3p- and 5p-derived miRNAs. The novel miRNAs based on their precursors were identified using the MiRDeep2 [17]. Clean data were imported into the miRDeep2 software, and the novel miRNAs were finally obtained by entering the running command (mapper.pl input.fa -c -j -l 18 -m -p genome.fa -s reads_collapsed.fa -t reads_collapsed_vs_genome.arf -v and miRDeep2.pl reads_collapsed.fa genome.fa reads_collapsed_vs_genome.arf miRNA.fa mature_other_miRNA.fa hairpin.fa)

The expression level of miRNAs was normalized by TPM (transcript per million) method. The differentially expressed miRNAs (DEmiRNAs) were selected with the thresholds of q-value < 0.05 and |log2 (foldchange)| > 1. The miRanda [18] and RNAhybrid software tools were used to predict the target of DEmiRNAs by comparing the candidate miRNA with the 3′-untranslated region (UTR) sequence of mRNAs. GO enrichment and KEGG pathway enrichment analysis of DEmiRNA targets were respectively performed using R based on the hypergeometric distribution. The top 30 GO terms and the top 20 of KEGG enrichment were considered to be enriched by targets.

### 2.7. Construction of DEmiRNA–mRNA Regulatory Network

The targets of DEmiRNAs were predicted using miRanda, RNAhybrid, and PITA software tools. A total of 100 mRNAs were predicted by taking the intersection of targets predicted by these three software tools and miRNA–mRNA interaction pairs were obtained. To identify the potential miRNA–mRNA interaction relationship, the Cytoscape 3.7.2 software was used to visualize the miRNA–mRNA regulatory network by importing miRNA–mRNA interaction pairs data into the Cytoscape software, which shows the inverse correlation of the miRNA and target expression.

### 2.8. Validation of miRNA and mRNA by Real-Time Quantitative PCR

To validate the RNA-seq data, three miRNAs were randomly selected for RT-qPCR confirmation with ChamQ™ universal SYBR^®^ RT-qPCR master mix (Vazyme, China). Total RNAs were extracted using RNA Isolater Total RNA Extraction Reagent (Vazyme, China). For miRNA determination, 1 µg of total RNA was reversed using the miRNA 1st Strand cDNA Synthesis Kit (by stem-loop) (Vazyme, China) with miRNA-specific stem-loop primers. For gene quantification, the cDNA was prepared using HiScript^®^ III All-in-one RT SuperMix Perfect for RT-qPCR (Vazyme, China) with the gDNA wiper. The PCR was conducted with a StepOnePlus™ Real-Time PCR System (Applied Biosystems, Thermo Fisher Scientific, Waltham, MA, USA) under the following conditions: 95 °C for 30 s, followed by 40 cycles of 95 °C for 10 s and 60 °C for 30 s. The specificity of each primer pair was ensured by analyzing the melting curve. Experiments were performed with three independent biological replicates and technical replicates. The relative quantity of miRNAs and mRNAs was analyzed by RT-qPCR and β-actin was used as the reference gene. All primers were synthesized by the Sangon Biotech Co., Ltd. (Shanghai, China) (Table S1). Data were analyzed by the 2^−ΔΔCT^ method.

### 2.9. Synthesis of dsRNA, RNA Interference and Viral Infection

Synthesis and microinjections of dsRNA were performed as previously described [19]. Briefly, target fragments of GFP (green fluorescent protein) and HaeIF3-S7 for dsRNA synthesis were amplified using specific primers. The PCR products were sub-cloned into the 5 min TA/Blunt-Zero Cloning Kit (Vazyme, China) and used as templates for target sequence amplification. By using a T7 RNAi Transcription Kit (Vazyme, China), the dsRNAs were synthesized in vitro with specific primers fusing the T7 promoter at the 5′ end and dissolved in DEPC water. The purity and yield of the dsRNA were checked on a 1.0% agarose gel and a spectrophotometer, respectively. The primers used to synthesize dsRNA are shown in Table S2.

In the knockdown experiment, newly molted fifth-instar larva was incubated for 30 min on ice, then injected with 10 μg single dsRNA solution (*ds GFP* and *ds HaeIF3-S7*) using a 10 µL microsyringe (Hamilton) in their last leg. Besides, an equivalent volume of DEPC was injected for the control.

The newly molted fifth-instar larvae injected with dsRNA solution were fed with fresh artificial diets for 4 h. The above larvae, after overnight starvation, were then fed with artificial diets coated with an MbMNPV suspension (OBs; 1 × 10^7^ OBs/larva). Upon consumption of the diets, the larvae were fed with fresh diets without exposure to any pathogens. Subsequently, three larvae infected with mock and MbMNPV were randomly selected at 24 h and 48 h, and the tissues of the fat body were dissected. To investigate the RNAi efficiency, RT-qPCR analysis was performed for the control group. To reveal the impact of RNAi HaeIF3-S7 on MbMNPV infection, the relative expression of polyhedrin was performed by RT-qPCR for the mock-infected and MbMNPV-infected groups.

### 2.10. Data Analysis

All the data in this study are presented as the means ± SEM. The statistical significance was determined by Student’s t-test for unpaired comparisons between two different groups, and *p* < 0.05 was regarded as statistically significant. All statistical analyses were performed with Prism software.

## 3. Results

### 3.1. MbMNPV Infection Caused Significant Reduction in Larval Wet Weight

**Figure 1:**
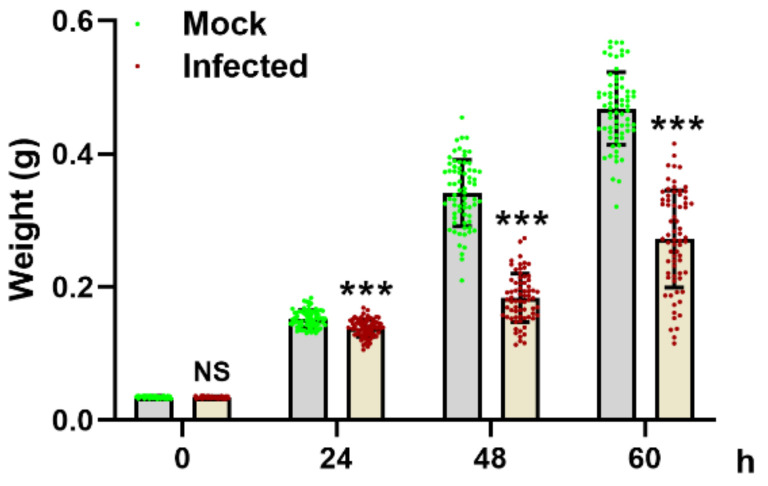
Effect of MbMNPV infection on body weight of *H. armigera*. ns, *p* > 0.05; ***, *p* < 0.001; *p* < 0.05 indicated the significant difference (Student’s *t*-test). Each experiment was performed in three replicates, and data are shown as mean ± s.e.m.

The wet weight of the mock-infected and MbMNPV-infected larvae was evaluated. Compared with the mock-infected group, the wet weight of the MbMNPV-infected larvae had a significant reduction (Figure 1). With the extension of infection time, despite the increase in larval weight, the wet weight was still significantly lower than that of the mock-infected group. This indicated that MbMNPV infection inhibited the growth of *H. armigera*.

### 3.2. Verification of the MbMNPV-Infected H. armigera

**Figure 2:**
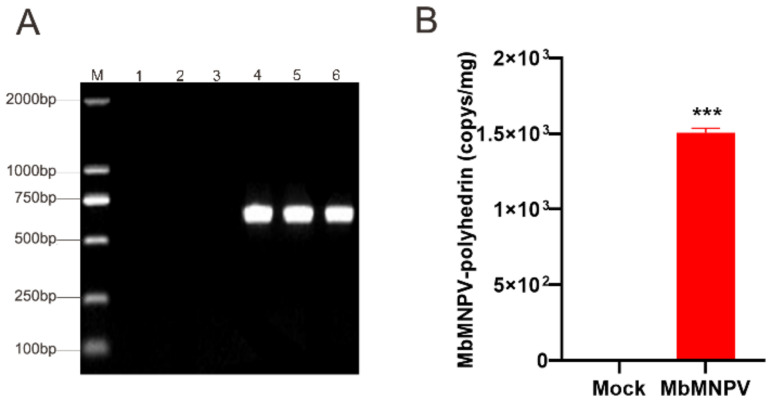
Verification for infection of *H. armigera* with MbMNPV. (**A**) PCR verification of polyhedrin gene of mock-infected and MbMNPV-infected larvae. Note: 1~6 are the amplification of polyhedrin gene in CK-1, CK-2, and CK-3 and MbMNPV-1, MbMNPV-2, MbMNPV-3. (**B**) The copy number of MbMNPV in the mock-infected and MbMNPV-infected larvae fat body. *** *p* < 0.001.

To verify the MbMNPV infection, the fat bodies of mock and MbMNPV-infected larvae were extracted and the infection was verified by PCR amplification of the polyhedrin gene. Our results indicated that the *H. armigera* were infected with MbMNPV successfully, while the control group was not infected with the virus (Figure 2A,B). The positive samples were used for further analysis.

### 3.3. MiRNA Sequencing and Data Analysis

**Figure 3:**
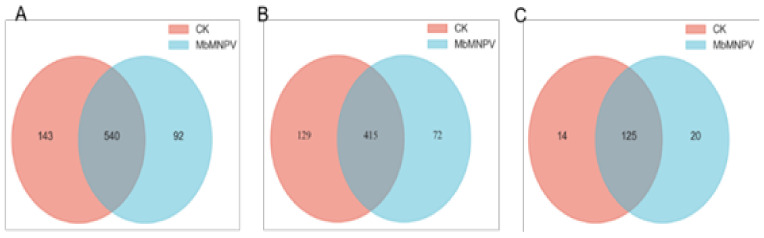
MiRNAs distribution in samples. (**A**) The distribution of total miRNA in the two samples. (**B**) The distribution of known miRNA in the two samples. (**C**) The distribution of novel predicted miRNA in the samples.

**Figure 4:**
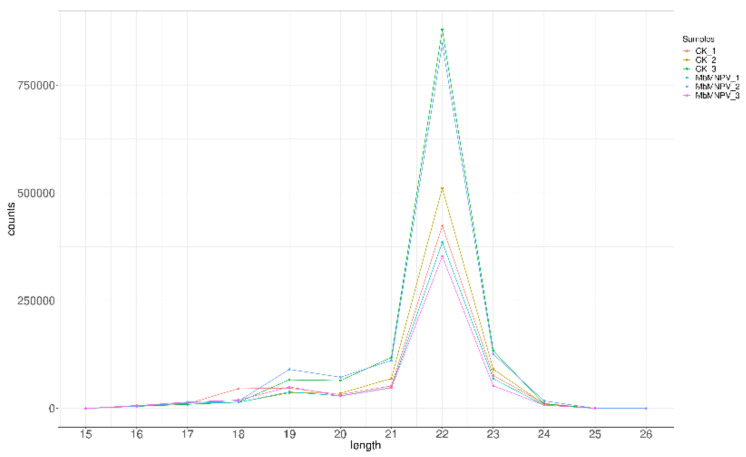
Length distribution of known miRNA.

Six small RNA libraries (mock-infected and MbMNPV-infected larvae, three repeats labeled as CK-1, CK-2, and CK-3, and MbMNPV-1, MbMNPV-2, and MbMNPV-3, respectively) were sequenced and the quality of the sequencing data was analyzed (Table 1). Clean reads were distributed at 23.28 M~24.12 M, with Q30 of 95.94~96.98%. A total of 775 miRNAs were identified, including 616 known miRNAs and 159 novel miRNAs (Figure 3). The length distribution of the known miRNA in all samples is shown in Figure 4. The results showed that most of the miRNA reads were 22 nt in size.

### 3.4. Functional Analysis for Targets of DEmiRNAs

**Figure 5:**
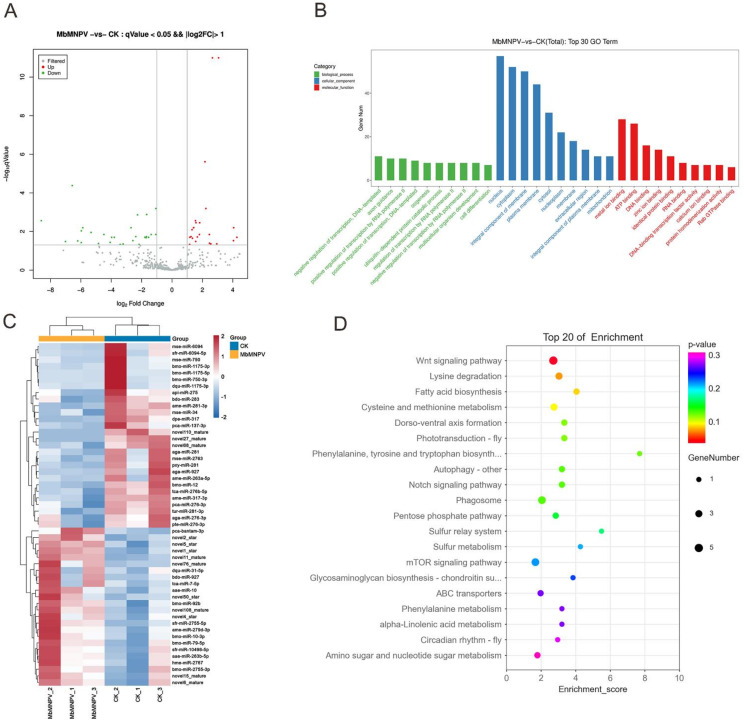
Screening and enrichment analysis of DEmiRNAs in MbMNPV-infected group compared with control group. (**A**) The volcano map of different miRNA. (**B**) Top 30 significantly enriched GO terms of predicted targets for DEmiRNAs in molecular function, cellular components, and biological process. (**C**) Hierarchical clustering analysis (heatmap) for DEmiRNAs. (**D**) Top 20 significantly enriched KEGG analyses of predicted targets for DEmiRNAs.

The DEmiRNAs between the MbMNPV-infected group and the control were screened based on TPM values (q < 0.05 and |log2 (foldchange)| > 1). A total of 52 miRNAs were differentially expressed, including 24 upregulated and 28 downregulated miRNAs (Figure 5A). The volcanic and heat maps (Figure 5B) of the normalized expression of miRNAs were generated and the expression patterns of the DEmiRNAs in the infection process were analyzed.

GO and KEGG analyses for the targets of DEmiRNAs were performed to investigate the function of DEmiRNAs (Figure 5C,D). GO analysis indicated that the predicted targets were mainly enriched in the following terms: positive regulation of transcription by RNA polymerase II (GO:0045944), ubiquitin-dependent protein catabolic process (GO:0006511), regulation of transcription by RNA polymerase II (GO:0006357), under biological process category; nucleus (GO:0005634), cytoplasm (GO:0005737), integral component of membrane (GO:0016021), under cellular component category; ATP binding (GO:0005524), DNA binding (GO:0003677), Rab GTPase binding (GO:0017137), under molecular function category (Figure 5C). In the KEGG pathway analysis, the Wnt signaling pathway (ID: haw04310), phagosome (ID: haw04145), mTOR signaling pathway (ID: haw04150), and cysteine and methionine metabolism (ID: haw00270) were significantly enriched (Figure 5D) (Table S3).

### 3.5. MiRNA Target Prediction and miRNA–mRNA Interaction Network

**Figure 6:**
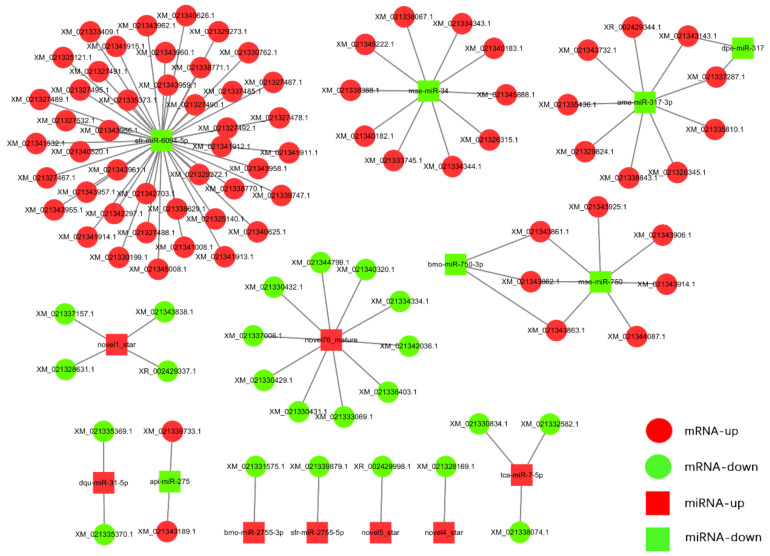
Interaction networks of differentially expressed miRNAs and putative target mRNAs in fat body of MbMNPV-infected *H. armigera*. The square nodes and circular nodes represent miRNAs and mRNA, respectively. Downregulated and upregulated RNAs are indicated by green and red colors, respectively.

The targets of 52 DEmiRNAs were predicted using miRanda, RNAhybrid, and PITA software. A total of 100 mRNAs were predicted by taking the intersection of targets predicted by these three software tools, which were associated with 15 DEmiRNAs, including 8 upregulated and 7 downregulated miRNAs. The inverse correlation of the miRNAs and target expression was analyzed and visualized by the Cytoscape software (Figure 6). Our analysis showed that except for novel4_star, novel5_star, sfr-miR-2755-5p, and bmo-miR-2755-3p for which only one target was predicted, other miRNAs could regulate multiple mRNAs. Four miRNAs (ame-miR-317-3p, mse-miR-34, sfr-miR-6094-5p, novel1-star) were predicted to have 9, 10, 44, and 4 mRNA targets, respectively.

### 3.6. Verification of DEmiRNAs by Real-Time Quantitative PCR

**Figure 7:**
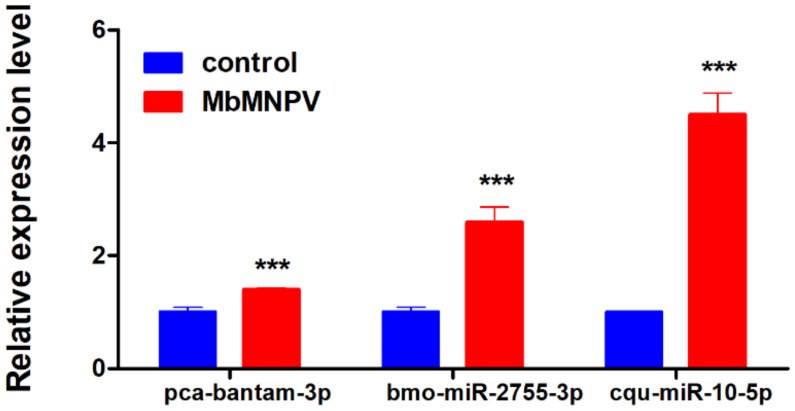
Relative expression of miRNAs in control and MbMNPV. ***, *p* < 0.001; *p* < 0.05 indicate significant difference (Student’s *t*-test). Each experiment was performed in three replicates, and data are shown as mean ± s.e.m.

Three miRNAs (pca-bantam-3p, bmo-miR-2755-3p, and cqu-miR-10-5p) were randomly selected from the DEmiRNAs to verify the sequencing data by RT-qPCR. The results showed that the expression of these three miRNAs in the MbMNPV-infected group was significantly upregulated compared with the control group (Figure 7). The RT-qPCR results were consistent with that of the sequencing results, indicating that the sequencing data was reliable.

### 3.7. Relative Expression of miRNAs in H. armigera Fat Body

Four DEmiRNAs (ame-miR-317-3p, mse-miR-34, sfr-miR-6094-5p, novel1-star) were selected based on the GO and KEGG analysis. Our sequencing results showed that the expression of ame-miR-317-3p, sfr-miR-6094-5p, and mse-miR-34 was downregulated and that of novel1-star was upregulated, at 72 h post-infection.

**Figure 8:**
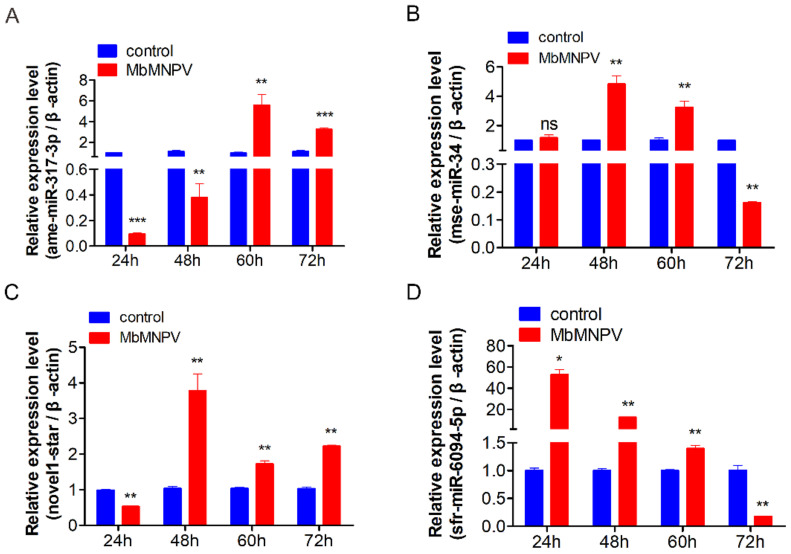
Relative expression of miRNAs at different time points in the control and MbMNPV-infected group. ns, *p* > 0.05; *, *p* < 0.05; **, *p* < 0.01; ***, *p* < 0.001, *p* < 0.05 indicate significant difference (Student’s *t*-test). Each experiment was performed in three replicates, and data are shown as mean ± s.e.m.

The relative expression of four miRNAs, at 24, 48, 60, and 72 h, in the virus infection group and control group was analyzed by RT-qPCR (Figure 8). The results showed that the relative expression of miR-317-3p in virus-infected fat bodies of *H. armigera* was low at 24 h. However, with the extension of virus infection time, its relative expression level gradually increased. The relative expression of miR-317-3p increased up to 60 hpi, which was different from the sequencing data. It may be caused by the host individual differences and sampling time (Figure 8A); the relative expression of mse-miR-34 was 4.8 times and 3.2 times higher at 48 h and 60 h in comparison to mock control, respectively. However, its expression at 72 h decreased in comparison to the control group (Figure 8B). The relative expression of miRNA novel1-star was significantly lower at 24 h, which increased at 48 h, 60 h, and 72 h (Figure 8C) in comparison to the mock control. The expression of sfr-miR-6094-5p was significantly upregulated at 24 h, and gradually the expression declined at 48 h and 60 h and was significantly downregulated at 72 h in comparison to the mock control (Figure 8D). The relative expression of mse-miR-34, novel1-star, and sfr-miR-6094-5p was consistent with the sequencing results.

The results proved that the miRNA expression profile of the host was indeed changed after virus infection at different treatment times, which indicated that these miRNAs may be involved in *H. armigera*–MbMNPV interaction. It is necessary to screen miRNA targets for follow-up research.

### 3.8. DEmiRNAs Inhibited Expression of Predicted Target Genes

By using the bioinformatics software, miRanda and RNAhybrid, the targets of sfr-miR-6094-5p were predicted. Based on GO and KEGG analysis, four miRNA–mRNA interaction pairs, eIF3-S7 (LOC110371347: XM_021327532.1), CG7583 (LOC110380909: XM_021341066.1), CG16901 (LOC110381560: XM_021341895.1) and btf314 (LOC110382609: XM_021343259.1), were selected for further analysis.

**Figure 9:**
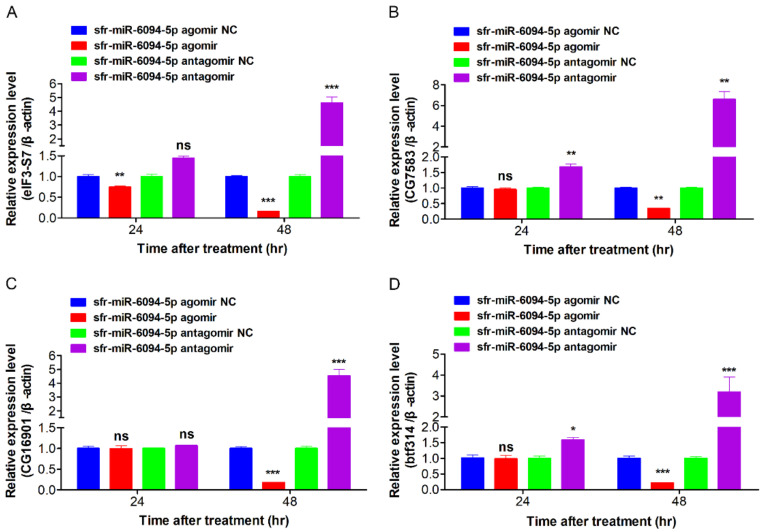
The relative expression of eIF3-S7 (**A**), CG7583 (**B**), CG16901 (**C**) and btf314 (**D**) after injection of sfr-miR-6094-5p agomir and antagomir. ns, *p* > 0.05; *, *p* < 0.05; **, *p* < 0.01; ***, *p* < 0.001; *p* < 0.05 indicate significant difference (Student’s *t*-test). Each experiment was performed in three replicates, and data are shown as mean ± s.e.m.

To verify the relationship of sfr-miR-6094-5p and predicted targets eIF3-S7, CG7583, CG16901, and btf314, overexpression and inhibition assay of sfr-miR-6094-5p were performed in vivo. The sfr-miR-6094-5p agomir or antagomir and their relative controls were injected into *H. armigera* larvae (fifth-instar, 1st day). Sequences for miRNA agomir and antagomir are shown in Table S4. The results are shown in Figure 9. Agomir injection of sfr-miR-6094-5p decreased the expression of eIF3-S7, CG7583, CG16901, and btf314 at 48 h post-treatment, while antagomir injection significantly increased these variables. In conclusion, the injection of sfr-miR-6094-5p agomir and antigomir could negatively regulate the expression of host gene eIF3-S7, CG7583, CG16901, and btf314 at 48 h, of which eIF3-S7 and CG7583 have been reported to be involved in host–virus interactions, indicating that sfr-miR-6094-5p may play a vital role in regulating host–virus interactions.

### 3.9. The Inhibition of sfr-miR-6094-5p on MbMNPV Infection

**Figure 10:**
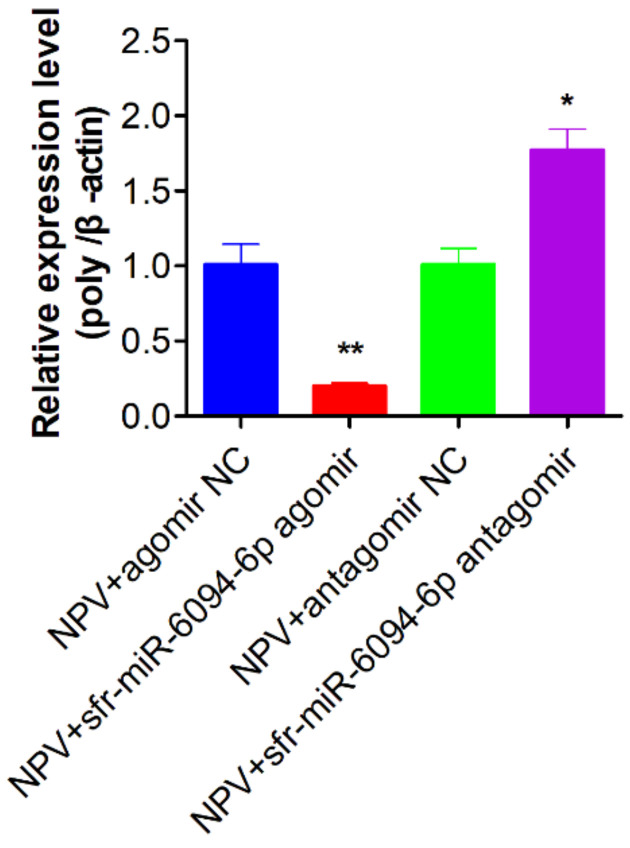
The relative expression of MbMNPV polyhedrin gene after injection of sfr-miR-6094-5p agomir and antagomir in the MbMNPV-infected larvae. *, *p* < 0.05; **, *p* < 0.01; *p* < 0.05 indicate significant difference (Student’s *t*-test). Each experiment was performed in three replicates, and data are shown as mean ± s.e.m.

To verify the impact of sfr-miR-6094-5p on MbMNPV infection, the sfr-miR-6094-5p agomir, antagomir, and their controls were injected into the MbMNPV-infected larvae, respectively. The relative expression of MbMNPV polyhedrin gene at 24 h post-infection, was detected by RT-qPCR. As shown in Figure 10, after injecting sfr-miR-6094-5p agomir, the expression of the polyhedrin gene in the experimental group was significantly downregulated and it was upregulated after antagomir treatment. These results indicated that sfr-miR-6094-5p negatively regulated MbMNPV infection in *H. armigera* larvae.

### 3.10. Knockdown of HaeIF3-S7 Inhibited the MbMNPV Infection

**Figure 11:**
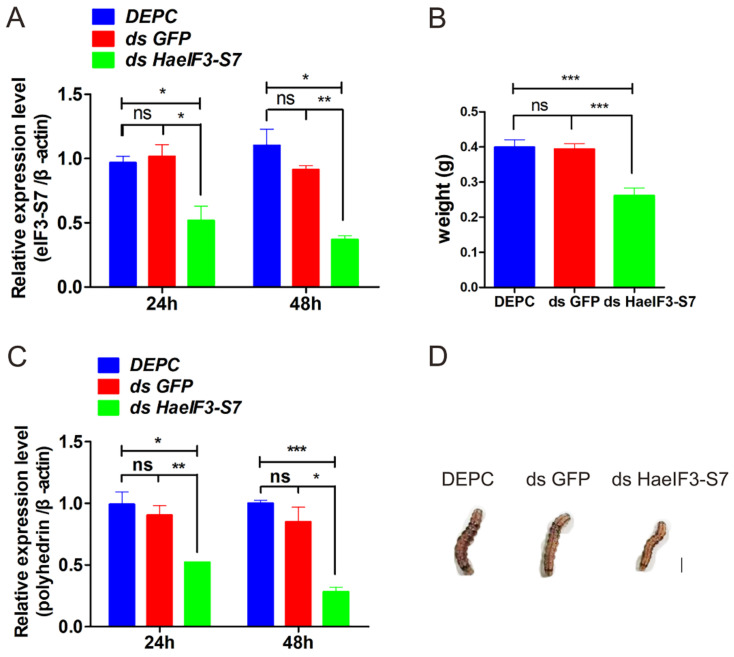
The effects of HaeIF3-S7 RNAi on the larvae. At 1d fifth-instar, larvae were injected with dsRNA of HaeIF3-S7 (ds eIF3-S7), dsRNA of green fluorescent protein (dsGFP), and DEPC (control). (**A**) The expression levels of the eIF3-S7 gene. (**B**) The expression levels of the polyhedrin gene. (**C**,**D**) The MbMNPV-infected larvae weight were analyzed. The standard error is represented by the error bar. ns, *p* > 0.05; *, *p* < 0.05; **, *p* < 0.01; ***, *p* < 0.001; *p* < 0.05 indicate significant difference (Student’s *t*-test). Each experiment was performed in three replicates, and data are shown as mean ± s.e.m.

As sfr-miR-6094-5p negatively regulates the target gene and MbMNPV infection, the effect of RNAi HaeIF3-S7 on MbMNPV infection was further investigated. After successful knockdown of eIF3-S7 (Figure 11A), we observed a significant decrease in MbMNPV replication (Figure 11B). Interestingly, at 48 h post-infection, the wet weight of the larvae with HaeIF3-S7 knocked down was downregulated (Figure 11C,D). In conclusion, our results show that the knockdown of eIF3-S7 inhibited MbMNPV infection.

## 4. Discussion

MiRNAs play pivotal roles in insect–baculovirus interactions [20]. Therefore, in the current study, we performed miRNA sequencing in the fat body of MbMNPV-infected *H. armigera.* A total of 52 DEmiRNAs were obtained, of which 24 were upregulated and 28 were downregulated. Of these miRNAs, bmo-miR-1175-3p [21], miR-12 [22], miR-92b [23], miR-34 [24], miR-927 [25], miR-281 [26], miR-927 [25], and miR-317 [27] have been reported in previous studies and known to be related to virus infection. Reports suggest that one specific miRNA can regulate the expression of multiple mRNAs and vice versa [28]. In the current study, the interaction networks of 8 upregulated and 7 downregulated miRNAs with their mRNA targets revealed similar patterns. The KEGG pathway enrichment analysis revealed that the predicted targets of DEmiRNAs were mainly enriched in the following signaling pathways: Wnt signaling pathway [29], mTOR signaling pathway [30], phagosome [31], and cysteine and methionine metabolism [32], which are related to virus infection.

In this study, ame-miR-317-3p, mse-miR-34, novel1-star, and sfr-miR-6094-5p were identified as DEmiRNAs in MbMNPV-infected *H. armigera*. It has been reported that the miR-34 family participates in immune response and regulates virus replication [24]. Among patients with liver fibrosis and hepatocellular carcinoma infected because of hepatitis B virus (HBV) infection, miR-34a-5p inhibits liver fibrosis by regulating the TGF-β1/Smad3 pathway [33]. It has been confirmed that miR-317 can be used as a negative regulator of toll immune response which affects *Drosophila* survival [34]. Since novel1-star is similar to pca-bandam-3p in clustering (Figure 5B), it may have functions similar to pca-bandam-3p.

In the current study, sfr-miR-6094-5p negatively regulates the expression of eIF3-S7, CG7583, CG16901, btf314, and against MbMNPV. eIF3-S7 and CG7583 have been reported to be involved in virus interactions. The eukaryotic initiation factor 3 (eIF3), a multi-subunit complex composed of 13 subunits (a-m), participates in translation initiation, termination, and ribosomal recycling [35], which may be the reason for the wet weight reduction in larvae. It has been reported that eIF3 is associated with virus replication. For example, overexpression of eIF3 can affect the replication of the yellow fever virus [36] and facilitates the replication of swine fever virus NS5A and the production of virus particles in offspring [37], which is consistent with our observation that the knockdown of Ha *eIF3-S7* inhibited MbMNPV replication. Our results showed that sfr-miR-6094-5p inhibited MbMNPV infection by negatively regulating the expression of eIF3-S7.

The C-terminal binding protein *CG7583* (CTBP) is enriched in Wnt signaling and Notch signaling pathways (haw04310, haw04330). The Wnt signaling pathway is involved in many biological processes [38]. It has been found that virus replication can be delayed by interfering with the interaction of CTIP and CTBP with miRNA [39]. Marek’s disease virus (MDV) encodes a major oncoprotein Meq, which is related to the interaction of CTBP [40]. CTBP also interacts with two oncoproteins, EBNA3A and EBNA3C, which are encoded by the Epstein Barr virus nuclear antigen 3C virus [41]. CG16901, as an RNA binding protein, is enriched in the Dorso-ventral axis formation signal pathway (haw04320). Zhang identified 17 differentially expressed proteins in studying the proteomics of the lymphoid organs of vibrio anguillarum shrimp, including a similar one to the squid CG16901-PC protein. The host gene btf314 is homologous to the transcription factor eIF3 and is enriched in the apoptosis fly pathway (haw04214), which plays a crucial role in maintaining the normal functioning of the body. Our results indicated that sfr-miR-6094-5p may also inhibit the MbMNPV infection by negatively regulating the expression of host CG7583, CG16901, and btf314, which needs to be further validated.

In conclusion, we profiled the differentially expressed miRNAs in the fat body of MbMNPV-infected *H. armigera* at 72 hpi and analyzed their interaction networks and putative functions. sfr-miR-6094-5p inhibits MbMNPV infection by negatively regulating the expression of eIF3-S7 in vivo. In the future, we will analyze the molecular mechanism of expression change of sfr-miR-6094-5p induced by MbMNPV, miR-6094-5p inhibiting the expression of eIF3 in *H. armigera*. This research can contribute a theoretical reference for insect pest control and shed light on further understanding of host–virus interactions.

## Figures and Tables

**Table 1:** Quality evaluation of sample sequencing data.

Sample	CK_1	CK_2	CK_3	MbMNPV_1	MbMNPV_2	MbMNPV_3
Raw reads	29.55 M	29.17 M	31.7 M	27.16 M	27.2 M	27.34 M
Sequences with Q20 Greater than 80%	23.92 M	24.13 M	23.32 M	23.83 M	23.97 M	24.16 M
Clean reads	23.88 M	24.08 M	23.28 M	23.79 M	23.93 M	24.12 M
Q30	95.94%	96.84%	96.71%	96.31%	96.79%	96.98%
GC content	55.31%	56.90%	56.94%	57.62%	57.20%	59.02%

## Data Availability

All relevant data are within the manuscript and its Supplementary Materials files.

## Supplementary Materials

The following supporting information can be downloaded at: https://www.mdpi.com/article/10.3390/v15010019/s1, Table S1: Primers used for real-time PCR; Table S2: Primers used for synthesizing dsRNA; Table S3: KEGG pathway enrichment analysis of DEmiRNAs targets; Table S4: Sequences for miRNA agomir and antagomir.
