# Supplementary material for: Integrated Analysis of MicroRNA and mRNA Expression Profiles in the Fat Bodies of MbMNPV-Infected Helicoverpa armigera
Source: Viruses. 2022 Dec 21;15(1):19. doi: 10.3390/v15010019 (PMC9861407; doi:10.3390/v15010019)
Supplement: Supplementary file 1 [file viruses-15-00019-s001.zip › viruses-2095745-supplementary.pdf]

Table S1. Primers used for real-time PCR.

Names	Sequences (5′–3′)
RT-pca-bantam-3p	GTCGTATCCAGTGCAGGGTCCGAGGTATTCG
	CACTGGATACGACATCAGC
F-pca-bantam-3p	GCGCGTGAGATCATTGTGAAA
RT-bmo-miR-2755-3p	GTCGTATCCAGTGCAGGGTCCGAGGTATTCG
	CACTGGATACGACAACAAG
F-bmo-miR-2755-3p	GCGCACCCTGTCAGACCATA
RT-sfr-miR-6094-5p	GTCGTATCCAGTGCAGGGTCCGAGGTATTCG
	CACTGGATACGACAGGTAC
F-sfr-miR-6094-5p	TCAGCGGTGGCCTGGG
RT-mse-miR-34	GTCGTATCCAGTGCAGGGTCCGAGGTATTCG
	CACTGGATACGACACAACC
F-mse-miR-34	GCGTGGCAGTGTGGTTAGCT
RT-novel1_star	GTCGTATCCAGTGCAGGGTCCGAGGTATTCG
	CACTGGATACGACTCTCAT
F-novel1_star	CCGCGTCATGCGAGGG
RT-ame-miR-317-3p	GTCGTATCCAGTGCAGGGTCCGAGGTATTCG
	CACTGGATACGACACTGAG
F-ame-miR-317-3p	CGTGAACACAGCTGGTGGTAT
F-actin	CACCGCTGAGAGGGAAATC
R-actin	CATACCCAGGAAGGAAGGC
R-universal	AGTGCAGGGTCCGAGGTATT

F-eIF3-S7	ATGCCTTACCAACCTTTCAGT
R-eIF3-S7	CCTCGTAGTGTCCACCAGATG
F-CG7583	GAGATGCCGATACTAAAAGACG
R-CG7583	GACCCAATACGGACGATGAT
F-btf3l4	AACACTTTCGCTATCACCG
R-btf3l4	ATGCCAGCCTCTTCAGC
F-CG16901	TAAAGTGATGGCTGCTGGCG
R-CG16901	TGTCAAATGGCATCTCAACCTC
F-polh	CGTAAACTGGAGCGGTAAA
R-polh	GACTTCGTGGGGCACATAG
eIF3-S7-T7-F	TAATACGACTCACTATAGGGTGAGGAAGAGGAAAGTGAGGTC
eIF3-S7-T7-R	TAATACGACTCACTATAGGGTCAGCGGTTTGTTGGGGTC

Table S2. Primers used for synthesizing dsRNA.

Names	Sequences (5′–3′)
eIF3-S7-T7-F	TAATACGACTCACTATAGGGTGAGGAAGAGGAAAGTGAGGTC
eIF3-S7-T7-R	TAATACGACTCACTATAGGGTCAGCGGTTTGTTGGGGTC

Table S3. KEGG pathway enrichment analysis of DEmiRNAs targets.

id	Term	ListHits	p-Value	Enrichment_score
haw04310	Wnt signaling pathway	5	0.035712781	2.710832899
haw00310	Lysine degradation	3	0.074366426	3.038986355
haw00061	Fatty acid biosynthesis	2	0.085721851	4.051981806
haw00270	Cysteine and methionine metabolism	3	0.094110229	2.749559083
haw04320	Dorso-ventral axis formation	2	0.118769207	3.347289318
haw04745	Phototransduction - fly	2	0.118769207	3.347289318
haw00400	Phenylalanine, tyrosine and tryptophan biosynthesis	1	0.123390405	7.698765432
haw04136	Autophagy - other	2	0.127468622	3.20781893
haw04330	Notch signaling pathway	2	0.127468622	3.20781893
haw04145	Phagosome	4	0.128903843	2.053004115
haw00030	Pentose phosphate pathway	2	0.154393253	2.851394604
haw04122	Sulfur relay system	1	0.168422664	5.499118166
haw00920	Sulfur metabolism	1	0.211168679	4.277091907
haw04150	mTOR signaling pathway	4	0.215456069	1.673644659
haw00532	Glycosaminoglycan biosynthesis - chondroitin sulfate / dermatan sulfate	1	0.231720412	3.849382716
haw02010	ABC transporters	2	0.269086722	1.974042418
haw00360	Phenylalanine metabolism	1	0.271250322	3.20781893
haw00592	alpha-Linolenic acid metabolism	1	0.271250322	3.20781893
haw04711	Circadian rhythm - fly	1	0.290255062	2.961063628
haw00520	Amino sugar and nucleotide sugar metabolism	2	0.307991188	1.790410566

Table S4. Sequences for miRNA agomir and antagomir.

Names	Sequences (5′–3′)
sfr-miR-6094-5p agomir	AUCAGCGGUGGCCUGGGGUACCU
sfr-miR-6094-5p antagomir	GUACCCCAGGCCACCGCUGAUUU
Negative control of agomir	UUCUCCGAACGUGUCACGUTT
Negative control of antagomir	CAGUACUUUUGUGUAGUACAA
